# Supplementary material for: Community composition of microbial microcosms follows simple assembly rules at evolutionary timescales
Source: Nat Commun. 2021 May 12;12:2891. doi: 10.1038/s41467-021-23247-0 (PMC8113234; doi:10.1038/s41467-021-23247-0)
Supplement: Supplementary file 1 — Supplementary Information [file 41467_2021_23247_MOESM1_ESM.pdf]

Supplementary information

Community composition of microbial microcosms follows simple assembly rules at evolutionary timescales

Authors: Nittay Meroz[1*], Nesli Tovi[1], Yael Sorokin[1], and Jonathan Friedman[1*]

Affiliation: [1]Department of Plant Pathology and Microbiology, The Hebrew University of Jerusalem, Rehovot, Israel.

The SI includes:

- Supplementary figures 1-16
- Supplementary table 1

Supplementary information

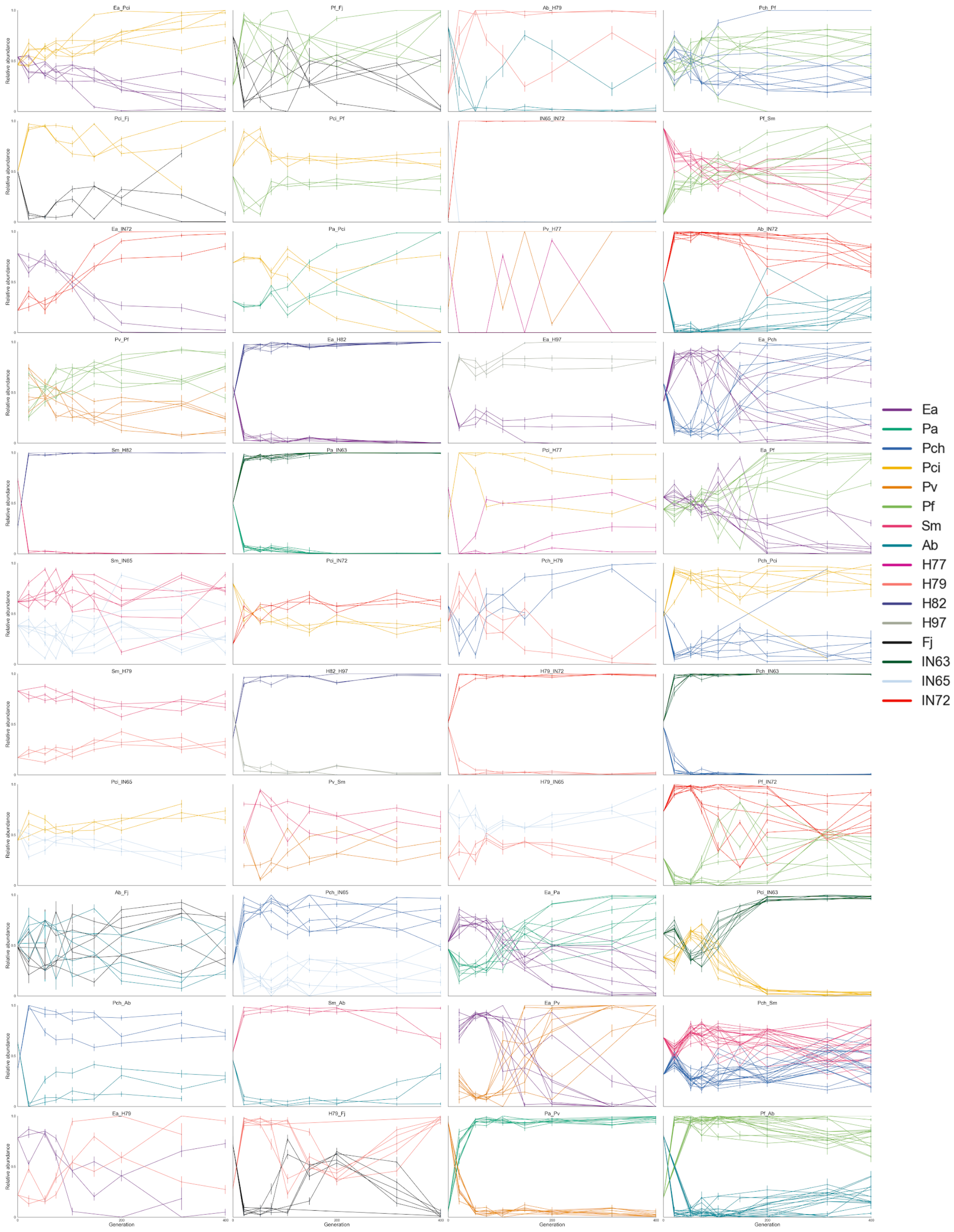

Figure S1: The community composition of all pairs throughout the experiment. *Colors denote the different species, and lines are replicates. The community composition was assayed by plating on agar plates and counting colonies, which are distinct for each species. Error bars represent the standard deviation of the posterior beta distribution of the fractions, based on colony counts in each replicate , Error bars were calculated as* $\sigma = \frac{\sqrt{p(1-p)}}{n+1}$, *where p is the observed species fraction* (colored dot) *and n is the total number of colonies counted for a given replicate.*

Supplementary information

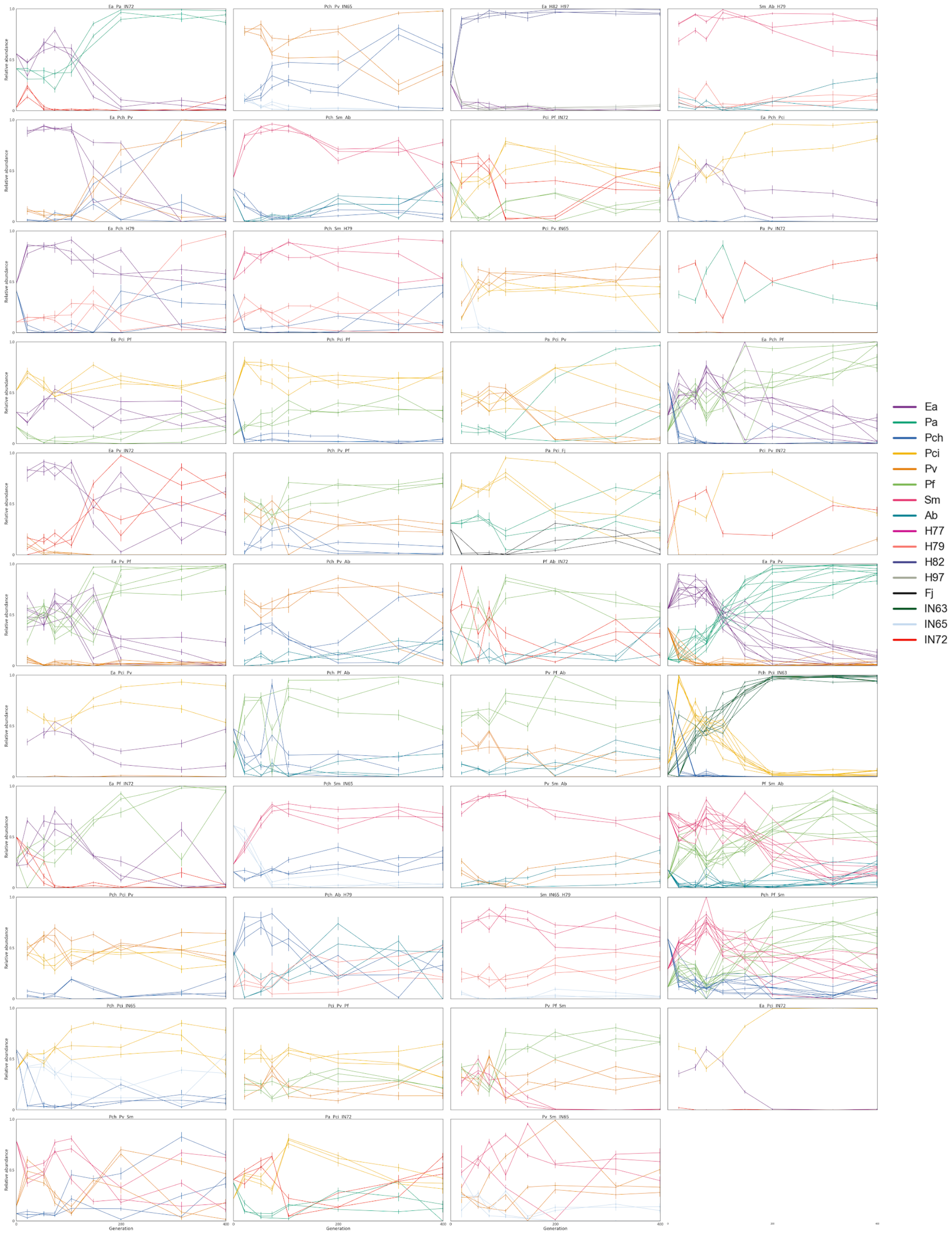

Figure S2: The community composition of all trios throughout the experiment. *Colors denote the different species, and lines are replicates. The community composition was assayed by plating on agar plates and counting colonies, which are distinct for each species. Error bars represent the standard deviation of the posterior beta distribution of the fractions, based on colony counts in each replicate , Error bars were calculated as* $\sigma = \frac{\sqrt{p(1-p)}}{n+1}$*, where p is the observed species fraction* (colored dot) *and n is the total number of colonies counted for a given replicate.*

Supplementary information

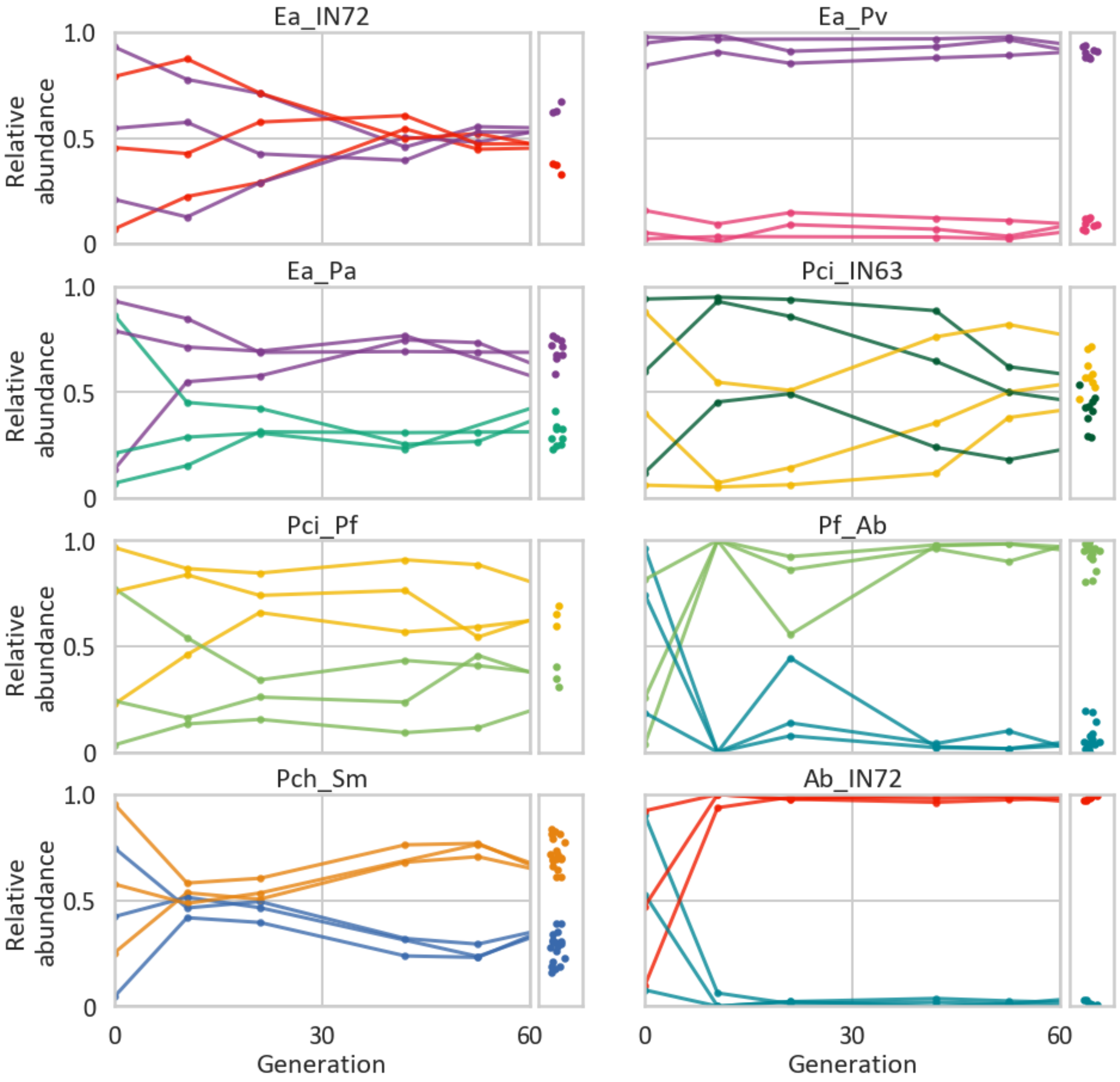

Figure S3: Pairs converge to the same composition from multiple initial fractions. *Line plots indicate the composition of pairs that started a ~60 generation competition experiment from three different initial conditions. Species were normalized to three initial fractions (0.1, 0.5, 0.9) by OD, thus a difference in the actual initial fractions as measured by CFU is to be expected. Colors denote different species. Narrow scatter plots near each line plot, indicate the fractions of the same pairs at the separate ~400 generation evolutionary experiment at generation ~70.*

Supplementary information

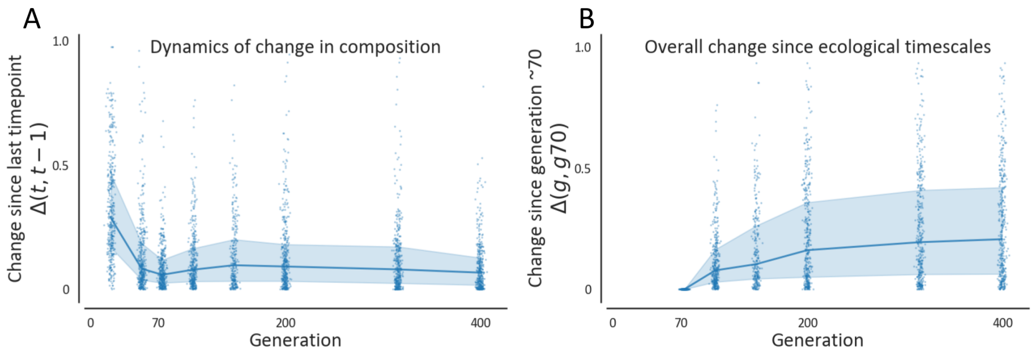

Figure S4: Dynamics of change in composition. *(A) Change in community composition across communities quantified as the Euclidean distance between the composition of each replicate at two subsequent time points normalized to the maximal distance between two communities composed of n species ($\sqrt{n}$,), denoted as $\Delta(t,\ t-1)$. (B) Change in community composition at evolutionary timescales measured as the Euclidean distance between the composition of each replicate in each time point and its composition at generation ~70 ($\Delta(g,\ g_{70})$). Generation ~70 is used here as the starting point of the evolutionary timescale since changes in most communities are less rapid after these timescales. For both A and B, lines denote the median, and shaded areas denote the interquartile range across all communities.*

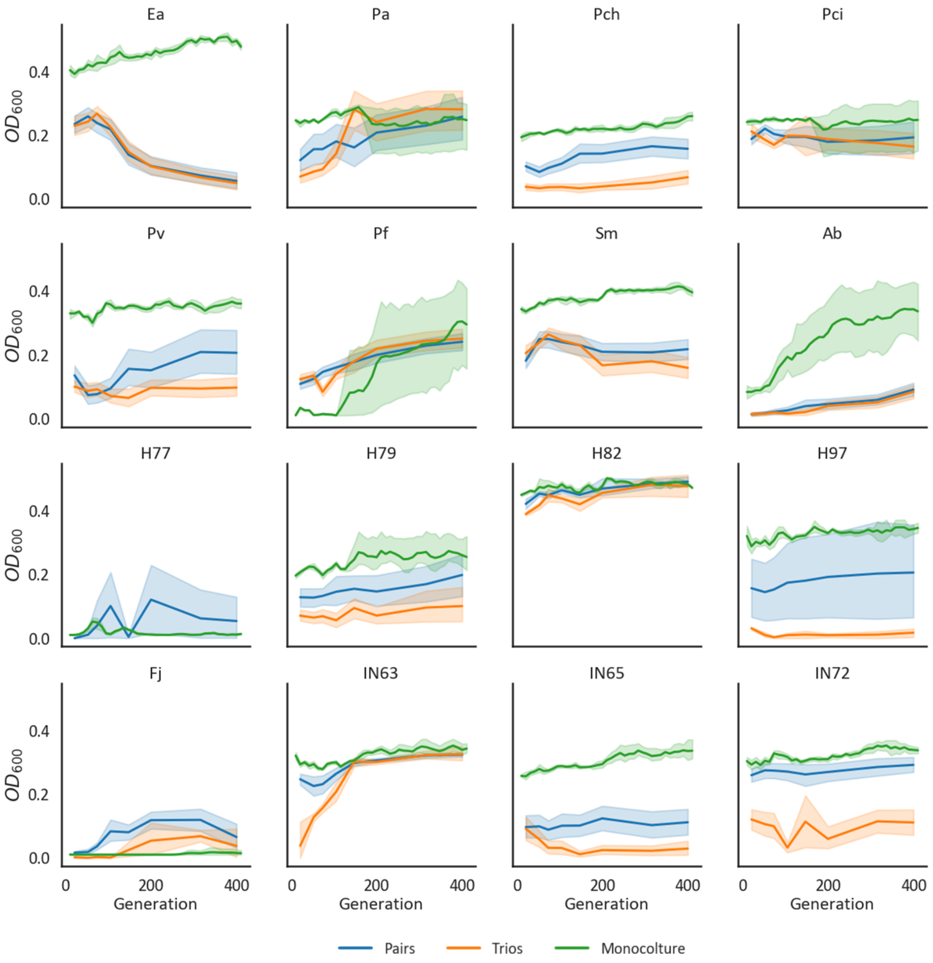

Figure S5: Absolute abundance of species changes throughout the experiment. *Absolute abundance is measured as the fraction of a species multiplied by the OD of the whole community. Blue and orange lines represent the median fractional OD of a species within all the pairs and trios respectively, and green line indicates the OD in monoculture. Shaded areas denote the .95 confidence interval and lines denote the means across all occurences of the specific species in the dataset.*

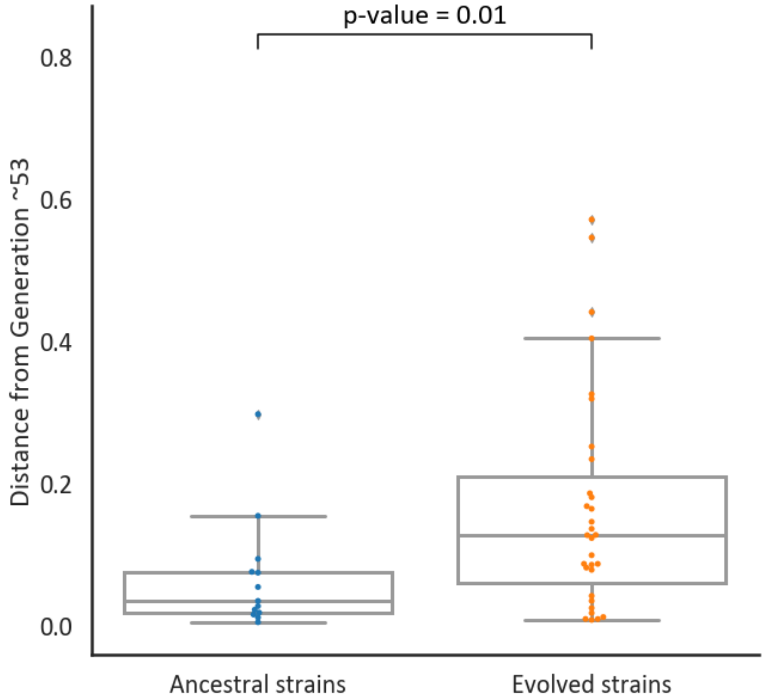

Figure S6. Pairs of evolved strains reach different compositions than those reached by their ancestors. *Data indicate the Euclidean distance normalized to the maximum distance ($\sqrt{2}$) of the composition reached by ancestral strains, and reisolated evolved strains in a short ~53 generation experiment, to the composition that was reached at the evolutionary experiment at generation ~53. Dots of ancestral strains indicate the mean of three technical replicates. Dots of evolved strains are averaged across two technical replicates, and 1-3 evolutionary replicates (communities that evolved in different wells). Boxes indicate the quartiles and whiskers are expanded to include values no further than 1.5X interquartile range. P-value indicates the p-value obtained by a one-sided Mann-Whitney U-test. Data includes 13 pairs of ancestral strains, and 30 pairs of coevolved derived strains.*

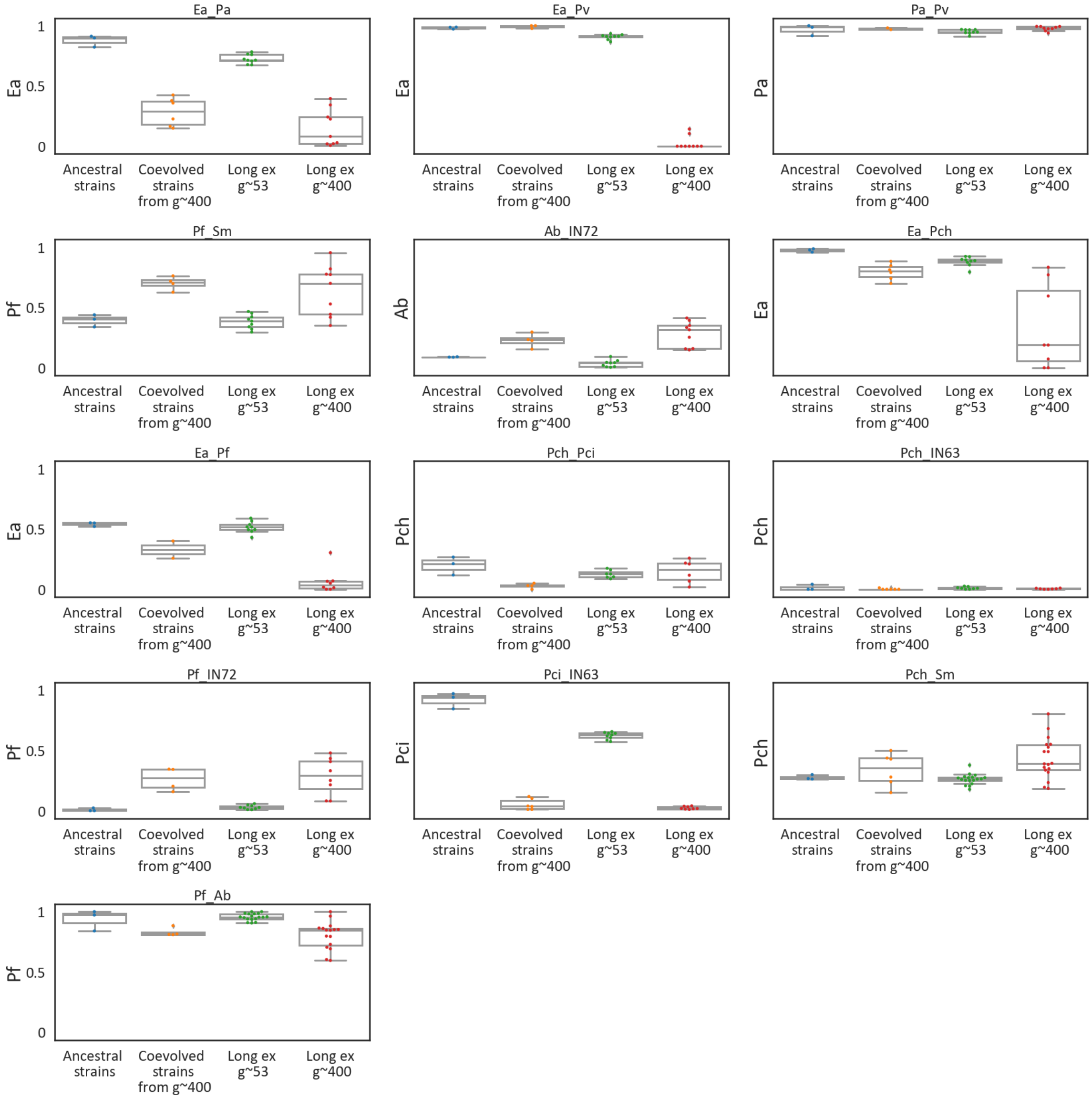

Figure S7. The composition reached by ancestral strains and coevolved strains after a ~53 generation experiment, and the composition of the same pairs at generations ~53 and ~400 of the long experiment. *Distributions represent the fraction of one of the species. Coevolved strains are strains that were reisolated from cocolture at generation ~400. The composition of coevolved strains was measured in two technical replicates and a varying number of evolutionary replicates. Boxes indicate the quartiles and whiskers are expanded to include values no further than 1.5X interquartile range*

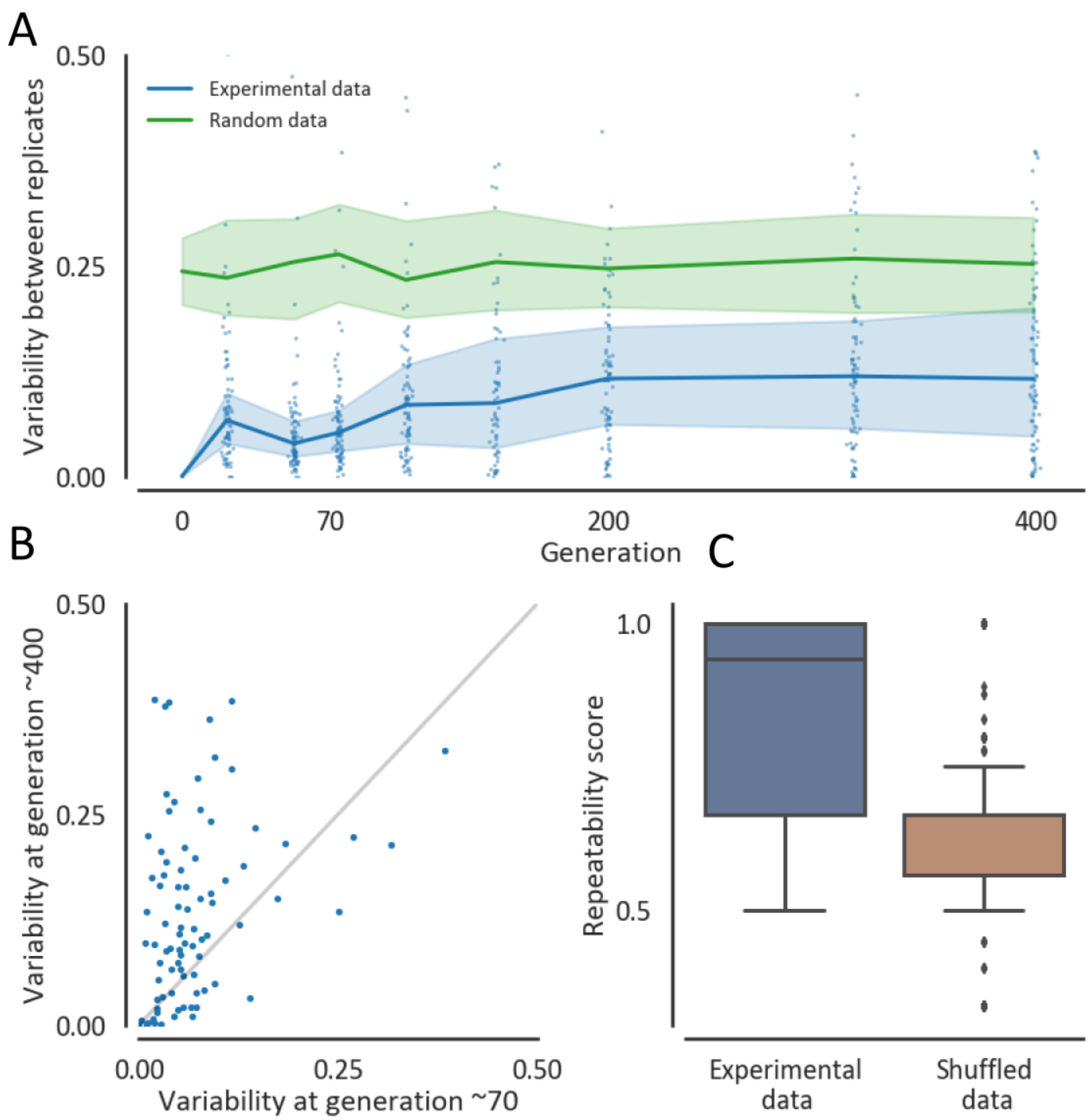

Figure S8: Repeatability of communities actress replicates. *(A) Variability in community composition between replicate communities is quantified by the mean Euclidean distance of each replicate from the medoid replicate normalized to the maximal distance between two communities composed of n species ($\sqrt{n}$,). Blue line and shaded-area are the median and interquartile range across all communities Dots denote the variability of specific pairs and trios across replicates. Green line and shaded-area are the median and interquartile range of randomly generated data with the same number of assemblages, replicates, and species as the experimental data (Methods). (B) Variability at generation ~400 against ~70, measured as the mean distance from. Each dot represents a community. (C) Distribution of repeatability scores of the experimental data and a random null model. Communities that had missing replicates and therefore had less than 3 replicates for either generation ~70 or ~400, were removed from this analysis. The brown boxes represent the distribution of 2000 iterations of a shuffled model, where the values of changes in relative abundances between generation ~70 and ~400 of all species are pooled and are subsequently randomly assigned (for pairs and trios separately) to any species in any community in the dataset. Boxes indicate the quartiles and whiskers are expanded to include values no further than 1.5X interquartile range.*

Supplementary information

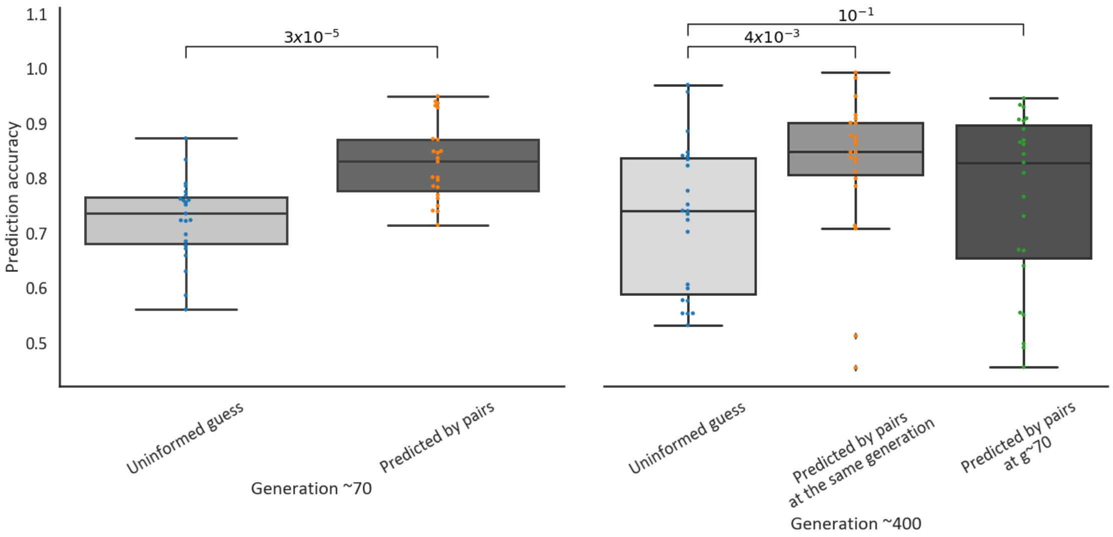

Figure S9: The composition of pairs predicts trios. *The distribution of prediction accuracies made by using the composition of pairs. The prediction accuracy is measured as* $1 - \frac{\Delta(Prediction, Observation)}{\sqrt{n}}$*, where* $\Delta(Prediction, Observation)$ *is the Euclidean distance between the prediction and the observation and* $\sqrt{n}$ *is the maximum distance between two communities with the same n species. Number above connectors indicate the p-values of a two-sided Mann-Whitney U test between the predictions and the null model. The null model is that all species have equal abundances in the trio. Boxes indicate the quartiles and whiskers are expanded to include values no further than 1.5X interquartile range.*

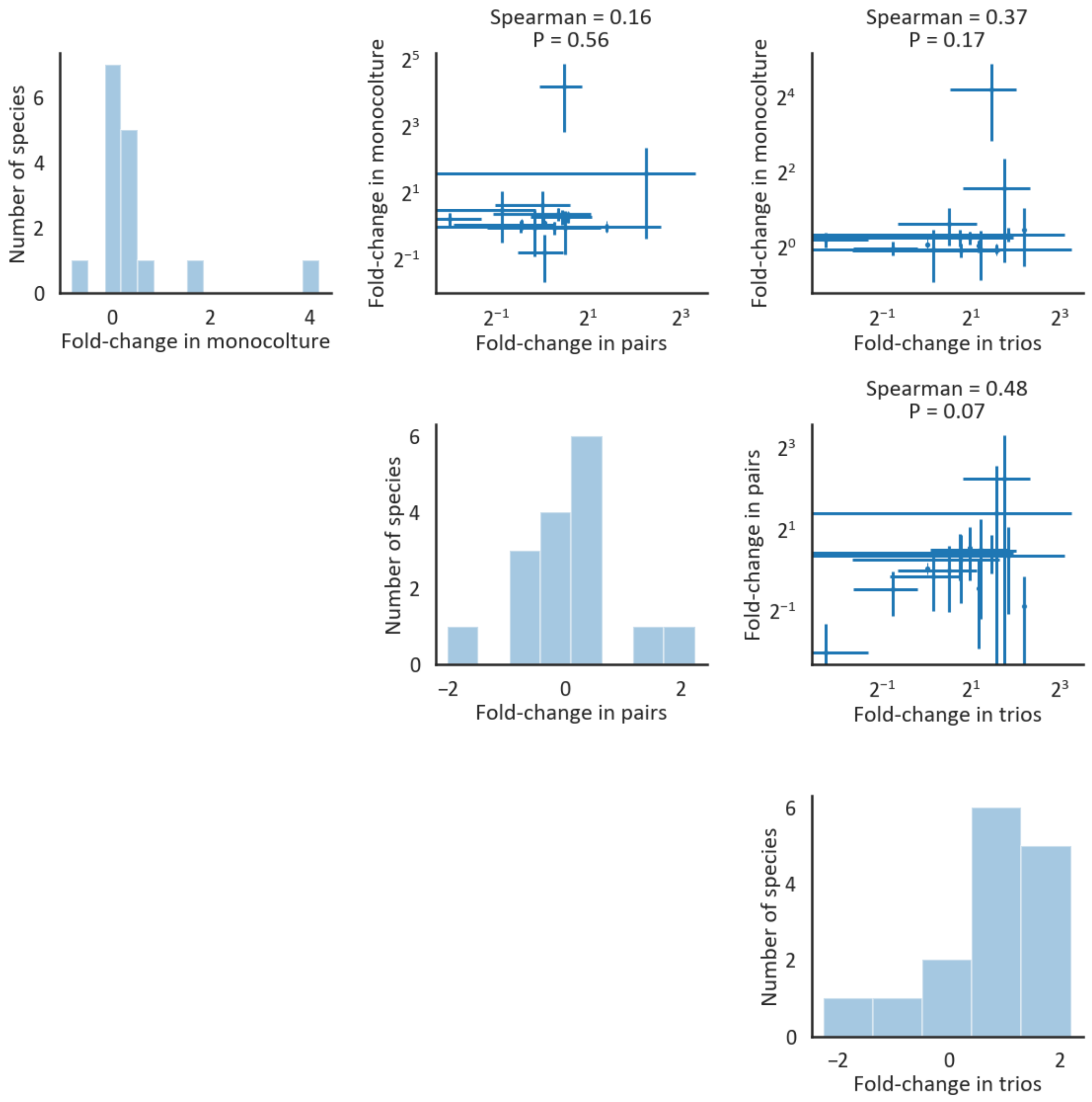

Figure S10: Changes in abundance that occur when species evolve in pairs and in trios are correlated with each other, but not with those that occur when species evolve in monoculture. *Fold-change in absolute abundance is measured as the log2 ratio of the fractional OD (fraction of a species multiplied by the OD of the community) of a specific species in a specific replicate at generations ~400 and ~70. Values are averaged for each species in all it's occurrences as a monoculture, in pairs, and in trios, separately. Histograms at the diagonal represent the distribution of average values for each one of the 16 species, in all communities with the same initial species' richness. Scatter plots compare the means of each species at two different community contexts. Error bars represent the standard deviation of a species' fold-change values for each treatment and are derived from a varying number of communities which is documented in Supplementary Data 1. Statistical results at the top of each scatter plot indicate the two-sided Spearman* ρ *and P-value.*

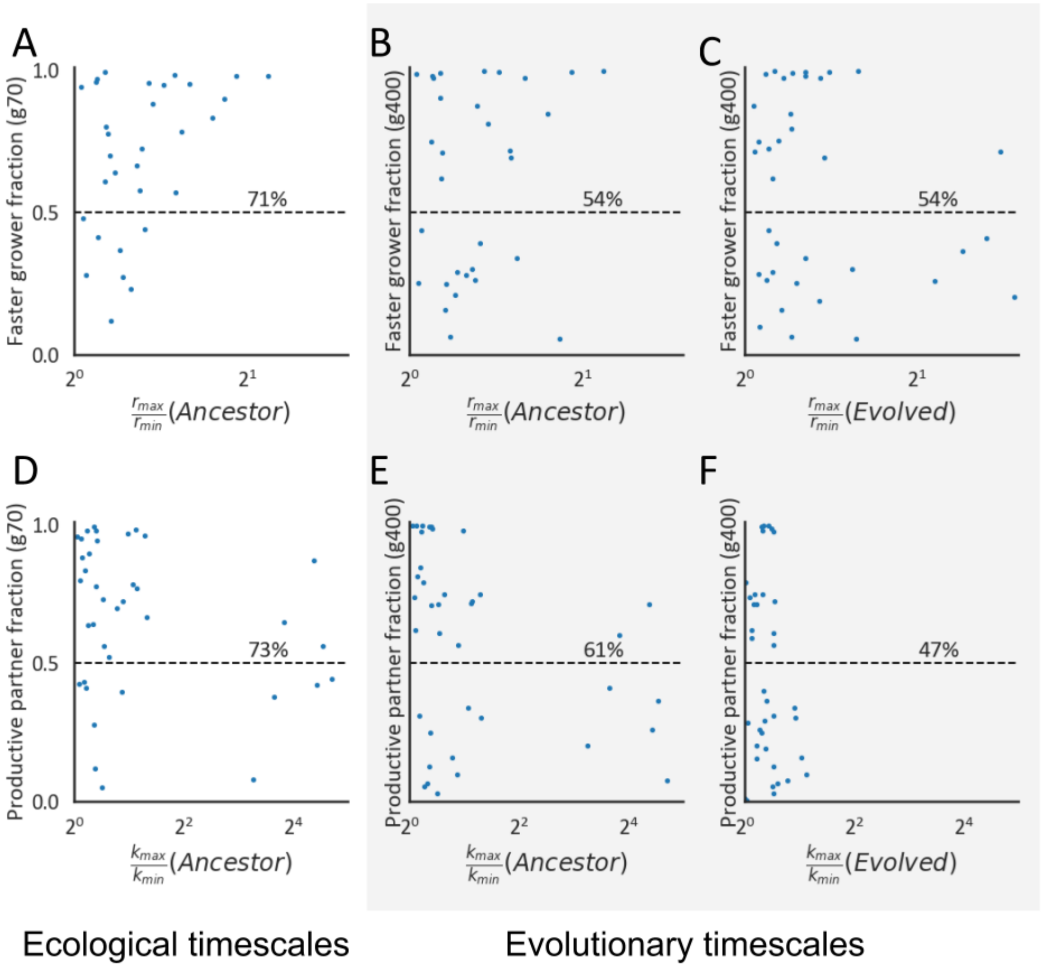

Figure S11: Growth of strains that evolved in monoculture does not predict the composition of pairs. *(A-C) The mean fraction of the species with the higher growth rate (A and B – ancestor, C – evolved) at generation ~70 (A) and generation ~400 (B, C) vs the ratio of the higher growth rate and the lower growth rate. (D-E) The mean fraction of the species with the higher carrying capacity (D and E – ancestor, F – evolved) at generation ~70 (E) and generation ~400 (E, F) vs the ratio of the higher carrying capacity and the lower carrying capacity. Percentages indicate the percentage of pairs above the 0.5 line, which correspond to the prediction that the species with the higher growth rate/carrying capacity is more dominant in a pairwise competition.*

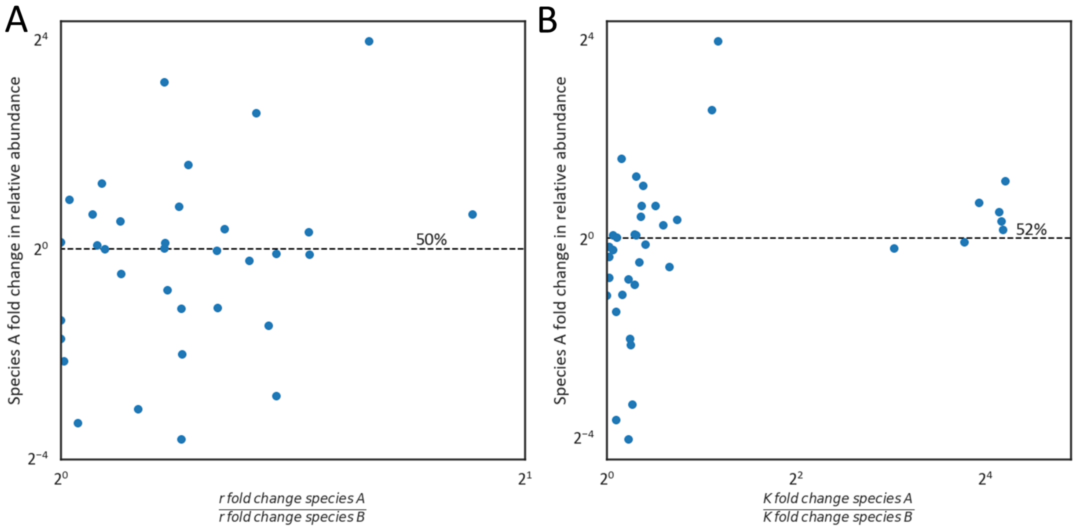

Figure S12: The increase in growth parameters of monocultures does not predict the increase in relative abundance in pairs. *(A) the fold difference in increase in growth rate between species A and species B against A's fold change in relative abundance where A is always the species that increased in growth rate by the larger factor. (B) the fold difference in increase in carrying capacity between species A and species B against A's fold change in relative abundance where A is always the species that increased in carrying capacity by the larger factor. The increase in carrying capacity is measured as the mean OD of a monoculture in generation ~400 divided by the mean OD of a monoculture in generation ~70. For both A and B the percentages indicate the accuracy of the prediction that the species that increased its growth rate of carrying capacity by the larger factor is also the one that increased its relative abundance.*

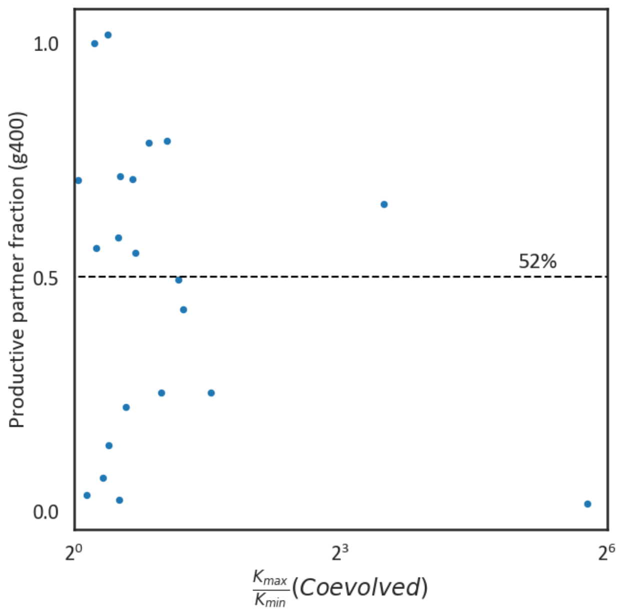

Figure S13: Growth of strains that evolved in coculture do not predict the composition of pairs. *The mean fraction of the species with the higher carrying capacity vs the ratio of the higher carrying capacity and the lower carrying capacity. Percentages indicate the percentage of pairs above the 0.5 line, which correspond to the prediction that the species with the higher growth rate/carrying capacity is more dominant in a pairwise competition. Data includes strains that were isolated from 21 pairs that evolved for ~400 generations (listed in Table S3).*

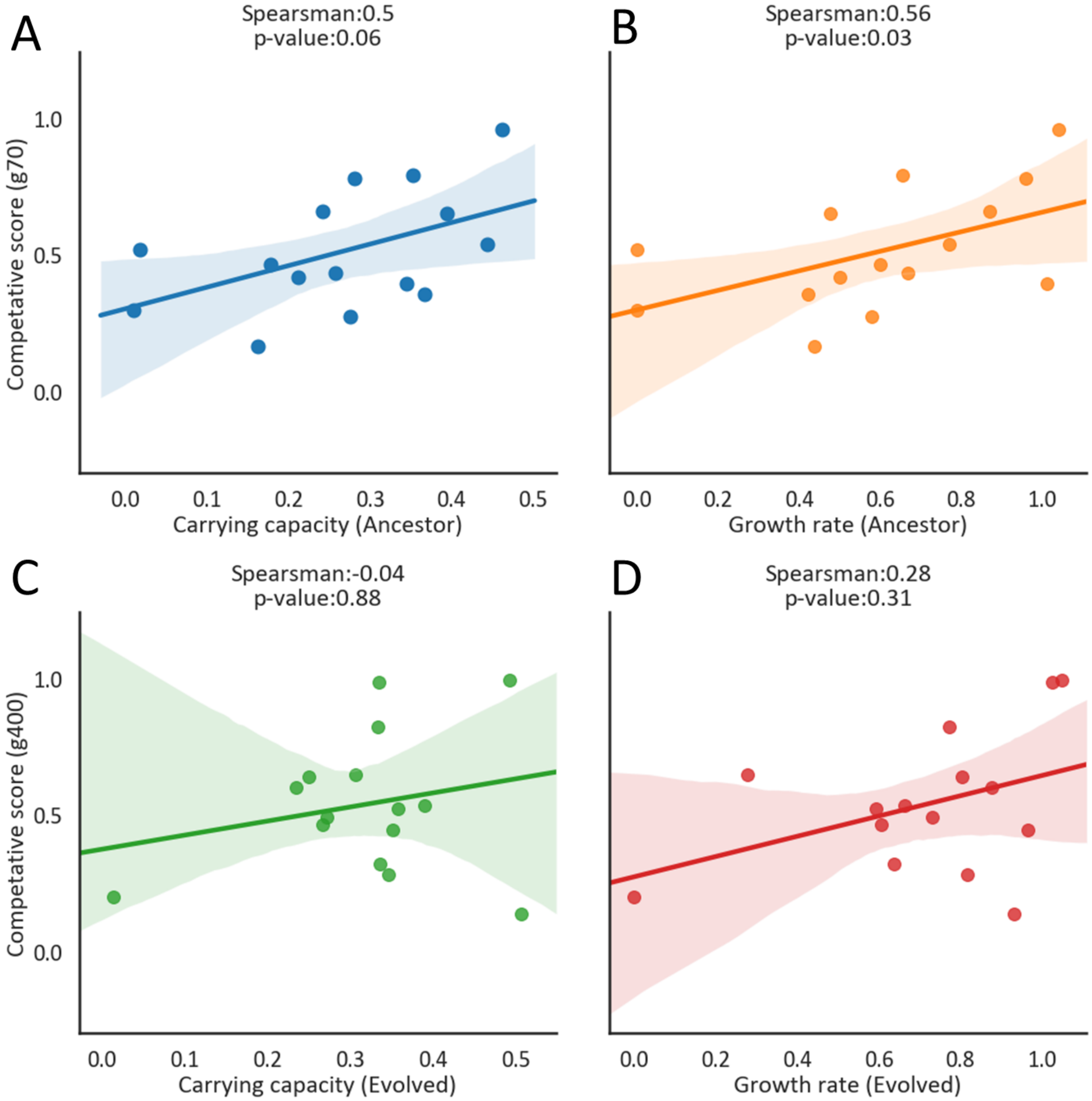

Figure S14: Correlations between species' growth abilities and species' competitive scores. *(A) The ecological competitive score, as the mean fraction of a species at generation ~70, against its carrying capacity as its mean OD at generation ~70. (B) The ecological competitive score against the mean effective growth rate of the ancestor (Methods). (C) The evolutionary competitive score, as the mean fraction of a species at generation ~400, against its carrying capacity as its mean OD at generation ~400. (D) The evolutionary competitive score against the mean effective growth rate of the monoculturally evolved strains. Lines denote a linear regression fit, and shaded areas denote the 0.95 confidence interval of the regression based on the data of 15 species. One species' growth (H77) was not measured and therefore was removed from this analysis. Statistical results at the top of each scatter plot indicate the two-sided Spearman ρ and p-value.*

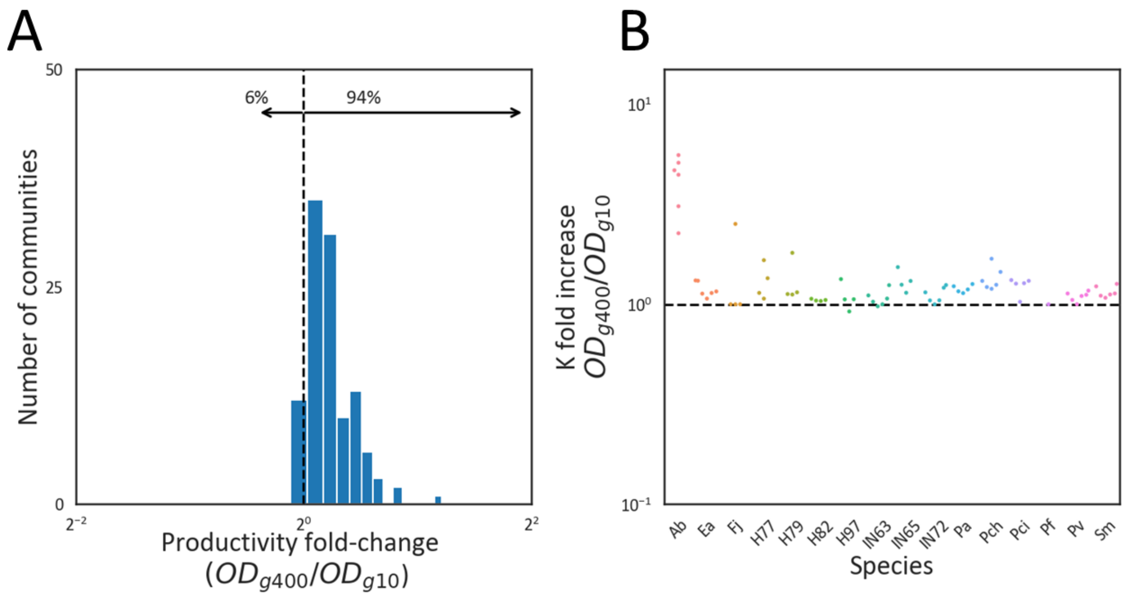

Figure S15: Most communities increase their productivity during the evolution experiment. *(A) A histogram of all communities' ratio between their productivity at generation ~400 to generation ~10 as the ratio between the OD they reached at the end of each growth cycle. To reduce noise, OD trajectories were smoothed by moving means with a window of three, and the OD of each unique assembly was averaged across all replicates. (B) the log increase in OD of the different species when grown in monoculture.*

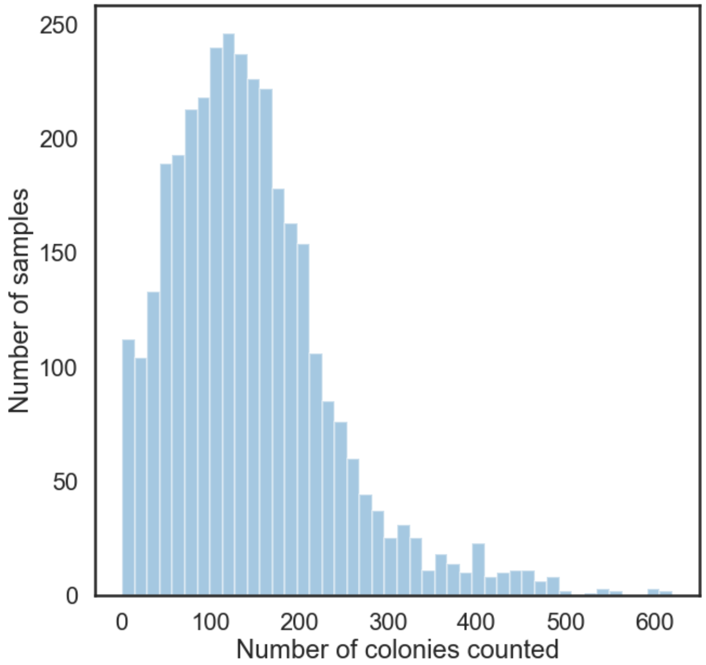

Figure S16: The distribution of the number of colonies counted to assay community composition throughout the experiment.

Supplementary information

Short Name	Species	source
Ea	*Enterobacter aerogenes ATCC 13048*	ATCC
Pa	*Pseudomonas aurantiaca ATCC 33663*	ATCC
Pci	*Pseudomonas citronellolis ATCC 13674*	ATCC
Pv	*Pseudomonas veronii ATCC 700474*	ATCC
Pch	*Pseudomonas chlororaphis ATCC 9446*	ATCC
Pf	*Pseudomans Fluroescens ATCC 506*	ATCC
Sm	*Serratia marcescens ATCC 13880*	ATCC
Ab	*Acinetobacter baylyi ATCC 3330*	ATCC
Fj	*Flavobacterium johnsonia strain UW101*	ATCC variant[39]
H77	*Arthrobacter phenanthrenivorans*	Tomato pot
H79	*Delftia lacustris*	Tomato pot
H82	*Pseudomonas putida*	Tomato pot
H97	*Pseudomonas pseudoalcaligenes*	Tomato pot
IN63	*Rhodococcus soli*	Wheat plot
IN65	*Pseudomonas alcaligenes*	Wheat plot
IN72	*Pseudomonas sp. BSP5*	Wheat plot

***Table S1: strains used in this study**. Environmental isolates are assigned to taxonomy by 16S sequence.*
