# Supplementary material for: Community composition of microbial microcosms follows simple assembly rules at evolutionary timescales
Source: Nat Commun. 2021 May 12;12:2891. doi: 10.1038/s41467-021-23247-0 (PMC8113234; doi:10.1038/s41467-021-23247-0)
Supplement: Supplementary file 3 — Description of Additional Supplementary Files [file 41467_2021_23247_MOESM3_ESM.pdf]

Description of Additional Supplementary Files

File Name: Supplementary Data 1
Description: Assemblages used for the coevolution experiment, along with the initial number of replicates of replicates for each, and the number of replicates that did not coexist for at least ~70 generations. Communities that did not coexists for ~70 generations, and contaminated replicates were removed from the analysis.

File Name: Supplementary Data 2
Description: Coevolved strains that were used for carrying capacity measurements. 'Coevolved with' column indicates the community context in which this strain has evolved in, and 'Evolutionary replicate ID' column indicates in which plate and well this strain has evolved in the ~400 generation experiment.
