# Supplementary material for: Community composition of microbial microcosms follows simple assembly rules at evolutionary timescales
Source: Nat Commun. 2021 May 12;12:2891. doi: 10.1038/s41467-021-23247-0 (PMC8113234; doi:10.1038/s41467-021-23247-0)
Supplement: Supplementary file 6 — Reporting Summary [file 41467_2021_23247_MOESM6_ESM.pdf]

nature research

Corresponding author(s): Jonathan Friedman

Last updated by author(s): Apr 13, 2021

Reporting Summary

Nature Research wishes to improve the reproducibility of the work that we publish. This form provides structure for consistency and transparency in reporting. For further information on Nature Research policies, see our Editorial Policies and the Editorial Policy Checklist.

Statistics

For all statistical analyses, confirm that the following items are present in the figure legend, table legend, main text, or Methods section.

n/a	Confirmed	
☐	☒	The exact sample size (*n*) for each experimental group/condition, given as a discrete number and unit of measurement
☐	☒	A statement on whether measurements were taken from distinct samples or whether the same sample was measured repeatedly
☐	☒	The statistical test(s) used AND whether they are one- or two-sided<br>*Only common tests should be described solely by name; describe more complex techniques in the Methods section.*
☒	☐	A description of all covariates tested
☒	☐	A description of any assumptions or corrections, such as tests of normality and adjustment for multiple comparisons
☐	☒	A full description of the statistical parameters including central tendency (e.g. means) or other basic estimates (e.g. regression coefficient) AND variation (e.g. standard deviation) or associated estimates of uncertainty (e.g. confidence intervals)
☐	☒	For null hypothesis testing, the test statistic (e.g. *F*, *t*, *r*) with confidence intervals, effect sizes, degrees of freedom and *P* value noted<br>*Give P values as exact values whenever suitable.*
☒	☐	For Bayesian analysis, information on the choice of priors and Markov chain Monte Carlo settings
☐	☒	For hierarchical and complex designs, identification of the appropriate level for tests and full reporting of outcomes
☐	☒	Estimates of effect sizes (e.g. Cohen's *d*, Pearson's *r*), indicating how they were calculated

Our web collection on statistics for biologists contains articles on many of the points above.

Software and code

Policy information about availability of computer code

Data collection	Optical density measurements were done using BioTek Instruments Gen 5 v3.09 software
Data analysis	Analysis was preformed with Python 3, analysis code is available at https://github.com/nittaym/Evo_assembly_rules

For manuscripts utilizing custom algorithms or software that are central to the research but not yet described in published literature, software must be made available to editors and reviewers. We strongly encourage code deposition in a community repository (e.g. GitHub). See the Nature Research guidelines for submitting code & software for further information.

Data

Policy information about availability of data

All manuscripts must include a data availability statement. This statement should provide the following information, where applicable:

- Accession codes, unique identifiers, or web links for publicly available datasets
- A list of figures that have associated raw data
- A description of any restrictions on data availability

Data is available at https://github.com/nittaym/Evo_assembly_rules

Field-specific reporting

Life sciences study design

All studies must disclose on these points even when the disclosure is negative.

Sample size	The study was designed to include a large number of different communities (initially 51 pairs and 51 trios, some were excluded - see next section). Sample size was determined by logistical constraints, rather than any specific statistical considerations. All assemblages consisted of subsets of a 16 species collection.
Data exclusions	Contaminated samples, and communities which did not coexist for at least ~70 generations (at least one species was extinct) were excluded from the analysis. Communities that did not coexist for at least ~70 generations were excluded because the study was designed to investigate the long term dynamics of change in community composition, therefore communities that did not coexist for long enough were irrelevant for this study.
Replication	Each community was replicated between 3-18 times. Supplementary Data 1 details the exact number of replicates for each community. Furthermore, 2 short ~50 generations experiments were done to replicate the community composition reached by pairs of ancestral and evolved strains and replicated the composition in most pairs successfully. The data of these experiments is shown on figures S3, S6, and S7.
Randomization	No grouping was done in this experiment
Blinding	As no grouping was done during this study, blinding in the data collection and in the data analysis is not relevant.

Reporting for specific materials, systems and methods

We require information from authors about some types of materials, experimental systems and methods used in many studies. Here, indicate whether each material, system or method listed is relevant to your study. If you are not sure if a list item applies to your research, read the appropriate section before selecting a response.

Materials & experimental systems

n/a	Involved in the study
☒	☐ Antibodies
☒	☐ Eukaryotic cell lines
☒	☐ Palaeontology and archaeology
☒	☐ Animals and other organisms
☒	☐ Human research participants
☒	☐ Clinical data
☒	☐ Dual use research of concern

Methods

n/a	Involved in the study
☒	☐ ChIP-seq
☒	☐ Flow cytometry
☒	☐ MRI-based neuroimaging
